# Effects of Different Physical Training Protocols on Metabolic Syndrome Indicators and the Activity of Butyrylcholinesterase in Adolescents: A Randomized Clinical Trial

**DOI:** 10.3390/metabo14080422

**Published:** 2024-07-31

**Authors:** Giuliano Roberto da Silva, Gerusa Dias Siqueira Vilela Terra, David Michel de Oliveira, Eduardo Vignoto Fernandes, Emerson José Zechin, Arthur Rizzi Soares, Dalton Muller Pessoa-Filho, Cassiano Merussi Neiva

**Affiliations:** 1 Department of Physical Education, Professor Edson Antônio Velano University, Alfenas 37132-440, Minas Gerais, Brazil; giumusc@gmail.com (G.R.d.S.);; 2 Postgraduate Program in Animal Bioscience, Federal University of Jataí, Jataí 75801-615, Goiás, Brazil; profdoliveira@ufj.edu.br; 3 Metabolism and Effort Physiology Laboratory, Faculty of Science, São Paulo State University, Bauru 17033-360, São Paulo, Brazil; emerson.zechin@unesp.br (E.J.Z.); arsoares@unaerp.br (A.R.S.); dalton.pessoa-filho@unesp.br (D.M.P.-F.); merussi.neiva@unesp.br (C.M.N.); 4 Postgraduate Program in Human Developmental and Technologies, São Paulo State University, Bauru 17033-360, São Paulo, Brazil; 5 College of Physical Education, Ribeirão Preto University, Ribeirão Preto 14096-900, São Paulo, Brazil; 6 Human Sports Performance Optimization Laboratory, Faculty of Science, São Paulo State University, Bauru 17033-360, São Paulo, Brazil

**Keywords:** cardiometabolic syndrome, physical activity, health of adolescents and youth, dyslipidemias, butyrylcholinesterase

## Abstract

Metabolic syndrome (MS) increases the risk of cardiovascular disease and affects children and adolescents. Butyrylcholinesterase (BChE) is an enzyme associated with obesity. The aim of this study was to investigate the effects of different physical training protocols on MS indicators and their relationship with BChE activity. This randomized clinical trial included 80 adolescents randomly assigned to 4 groups (CG: Control Group; ATG: Aerobic Training Group; STG: Strength Training Group; and CTG: Concurrent Training Group). The EFC, lipid profile, glycemia, waist circumference, and blood pressure were analyzed. With the exception of the CG, all the groups underwent training protocols for 12 consecutive weeks, 4 times a week, as follows: (ATG: 75% of heart rate on an electric treadmill; STG: 85% of 1 maximum repetition; CTG: 20 min of aerobic training at the same intensity as the ATG, and 20 min of resistance training in the same way as the STG). The training reduced MS-related biomarkers, such as the lipid profile, glycemia, waist circumference, and blood pressure. STG reduced BChE activity. The training methods led to improvements in the majority of the MS indicators. In addition, aerobic training significantly reduced BChE activity after a 12-week training protocol. The results suggest that different types of exercise can benefit MS.

## 1. Introduction

Metabolic syndrome (MS) is characterized by the clustering of pathophysiological abnormalities, such as central obesity, dyslipidemia, systemic arterial hypertension (SAH), insulin resistance, and diabetes—i.e., a set of cardio-metabolic risk factors, representing an important global public health problem that has attracted the attention of researchers worldwide due to its rapid growth [1].

The World Health Organization (WHO) points out that the global prevalence of metabolic syndrome in children and adolescents has reached high indices, around 36.8% of the world’s population [2]. When focusing on the child and adolescent populations, in parallel with the growth of obesity, the prevalence of MS in this age group has shown an alarming increase [3]. Systematic reviews have shown worldwide prevalences of MS among children and adolescents of 3.3% and of overweight/obesity of 29.2%, or 6 to 39%, as the identification of MS in children and adolescents is still a controversial issue [4,5].

According to the International Diabetes Federation (IDF) [6], the criteria for diagnosing metabolic syndrome in children and adolescents are: central obesity (waist circumference (WC) greater than 90 cm); triglycerides above 159 mg/dL; HDL-c below 40 mg/dL; blood pressure above 130 mmHg (systolic) and/or 85 mmHg (diastolic); and glucose levels above 100 mg/dL or known type 2 diabetes mellitus (recommended oral glucose tolerance test).

Early diagnosis of MS is essential [5] because of its multifactorial etiology and because each disease component is a risk factor for morbidity and mortality due to cardiovascular diseases, which represent the major cause of death in the world [7].

Among the traditional MS risk factors, hyperglycemia and insulin resistance particularly promote a pro-inflammatory state by increasing interleukin concentrations, tumor necrosis factor, reactive oxygen species, and advanced glycosylation, which are related to cellular and molecular damage and are closely linked to lipid metabolism, the etiogenesis of cardiovascular diseases, and increased chances of mortality [7].

Therefore, the discovery of biomarkers that can signal the presence of lipid imbalances that lead to the development of MS at an early stage seems to be an interesting path. Butyrylcholinesterase (BChE), a serum esterase and an enzyme of the cholinesterase group, may be one of these biomarkers [8]. Cholinesterase, including butyrylcholinesterase (BChE—E.C. 3.1.1.8), is present in the serum and low-density lipoproteins (LDL) of hyperlipidemic patients. This suggests a potential role of BChE in lipid transport, given its presence in LDL, the main transporter of cholesterol and other lipids, indicating the involvement of BChE in the regulation and metabolization of lipid metabolism, with relevance to hyperlipidemic conditions. The presence of BChE in these lipoproteins underlines its potential contribution to lipid-related metabolic disorders, such as hyperlipidemia, and emphasizes its importance in lipid metabolism and its clinical implications in metabolic disorders [9,10].

Although widely distributed in various tissues of the human body, the physiological role of BChE is still undefined. Some studies demonstrated a positive association between BChE activity and BMI, LDL-c, total cholesterol (TC), and triglycerides (TG). This suggests the hypothesis that it may be considered a new marker of atherosclerotic disease, which, together with other biomarkers, could improve the potential for cardiovascular risk assessment [11,12,13].

Hyperlipidemic [14] and obese individuals [15], especially those with abdominal obesity [16], presented higher levels of the BChE enzyme than normal-weight individuals. This enzyme has been related to some risk factors for cardiovascular diseases, such as obesity, changes in lipid metabolism, blood pressure, and insulin behavior [15]. Physical exercise, especially when performed regularly, has significant effects on reducing the activity of butyrylcholinesterase (BChE). According to Milano et al. [15], this enzyme, which is associated with lipid metabolism, is influenced by moderate to high intensity physical activity, in both obese adolescents and young men. The mechanisms responsible for the reduction in BChE activity are related to increased energy demand during exercise and increased use of lipids as an energy source, which can directly affect BChE activity, since it is involved in lipid metabolism [17]. In addition, physical exercise can also modulate the expression and activity of other enzymes and hormones involved in lipid metabolism, which indirectly influences the activity of BChE. These exercise-induced metabolic alterations contribute to an improvement in metabolic health and body weight control, suggesting that regular exercise may be an effective strategy for modulating the activity of BChE and reducing cardiovascular risk factors in obese and young individuals [18].

Given these facts, the initial concern that gave rise to this study was to analyze whether physical exercise interferes in any way with BChE activity, as well as with other MS indicators. The literature includes several studies that demonstrate, in isolation, the efficiency of exercise practice to control MS indicators. However, the type of exercise that is most effective in this control is still inconclusive, as mentioned in the study by Mann, Beedie, and Jimenez [19]. These authors suggest that there are no studies in the literature that clarify this problem by comparing different methods of exercise: aerobic, resistance, and concurrent.

Given such questions and concerns, the current study aimed to investigate the effects of three different exercise modalities (aerobic, resistance, and concurrent) on BChE activity and on MS indicators (WC, TC, TG, HDL-c, LDL-c, glycemia, and BP) in adolescents with MS.

## 2. Materials and Methods

### 2.1. Design, Research Location, and Participants

This randomized clinical trial was carried out in a University Hospital in the city of Alfenas, located in the state of Minas Gerais, southeastern Brazil. The city has a demographic density of 92.86 hab/km^2^, with 6.39% being young people who are aged between 15 and 19 years and are students from public schools [20]. The sample was estimated using a sample size simulator (G *Power^®^, version 3.1.9.2; Institute for Experimental Psychology in Dusseldorf, Germany), with type I and II errors set at α = 0.05 and β = 0.05, respectively, in order to obtain an effect size equal to or greater than 0.50. The calculation determined the need to collect data from 76 people. In this way, 80 adolescents aged 16.9 (±1.1) years, of both sexes, diagnosed with MS according to IDF criteria, were selected to participate in the study. This figure represents 5% more than the sample size calculation, to allow for any dropouts.

The inclusion criteria were as follows: being a student regularly enrolled in a public middle or high school in the municipality; aged 14 to 18 years at the beginning of the study; not practicing regular physical activity, sports, or other forms of workout or regular physical effort; and having MS as defined by the IDF [6].

For the participants under 18 years of age, the parents signed an informed consent form, while those aged 18 years or above signed the consent form themselves, which was also considered an inclusion criterion.

The exclusion criteria were as follows: Individuals who were under any form of treatment (pharmacological or otherwise) for MetS indicators; individuals with hypothyroidism, liver disease, protein-calorie malnutrition, kidney disease, or other diagnosed metabolic diseases; individuals with orthopedic or cardiovascular limitations to physical exercise; individuals using lipid-lowering or antihypertensive drugs; individuals with a medical diagnosis or whose parents reported delayed or precocious puberty; and those who were unwilling to participate in the entire protocol.

The sample comprised 42 female participants, with an average age of 15.7 ± 1.2 years and classified as being in maturational stages 4 and 5, and 38 male participants, with an average age of 16.9 ± 1.0 years and classified as being in maturational stages 3 and 4 [21]. The participants did not receive any kind of nutritional supplement or follow any specific diet.

The study was conducted in accordance with the Declaration of Helsinki, and approved by the Ethics Committee of Universidade José Rosário Vellano/UNIFENAS (protocol code CAAE 50765915.5.0000.5143 and was approved on 3 December 2015, under opinion no. 1348952). The project was registered with the Brazilian Clinical Trials Registry Platform (ReBEC) under the number RBR-2rhxcc8 (https://ensaiosclinicos.gov.br/rg/RBR-2rhxcc8), accessed 29 July 2024.

### 2.2. Procedures, Parameters, and Indicators for Analysis

At the beginning and end of the experimental protocol (after 12 weeks), the following parameters were collected: waist circumference in cm, measured with an inelastic tape accurate to 0.1 cm, applied above the iliac crest, parallel to the ground, with the individual standing with a relaxed abdomen, arms alongside their body, and feet together; blood pressure with the individual sitting at rest; and biochemical data (concentrations of total cholesterol (TC); high-density lipoprotein (HDL-c); low-density lipoprotein (LDL-c); triglycerides (TG); glycemia; and BChE activity).

According to Dietz et al. [22], although there are many methods for measuring cholinesterase activity (E.C. 3.1.1.8), few are adapted to recognize atypical alleles. In the present study, a rapid colorimetric method (Ellman method) was used to determine the activity of the enzyme BChE, with the specific substrate butyryl-thiocholine, which is hydrolyzed by the enzyme, producing a detectable product, thioclic acid. The rate of product formation is measured spectrophotometrically, over a given time interval, to determine enzyme activity, as described by Ellman et al. [23].

Blood samples for all humoral analyses employed in the study were undertaken in an appropriate room, in the morning, between 7:00 and 8:00 am, following an 8- to 12-h overnight fast, by phlebotomy via superficial puncture of the ulnar vein, in the forearm. Serum blood tests were performed in a single clinical laboratory. The participants were asked to maintain their habitual eating habits, unchanged, throughout the experiment period.

### 2.3. Intervention Protocols

After the initial analysis, the participants were divided into 4 experimental groups (20 participants in each group):

- Control Group (CG): 11 females with an average age of 15.2 ± 1.3 years and 9 males with an average age of 16.4 ± 1.3 years.
- Aerobic Training Group (ATG): 10 females with an average age of 15.4 ± 1.1 years and 10 males with an average age of 16.7 ± 1.1 years.
- Strength Training Group (STG): 10 females with an average age of 17.0 ± 1.3 years and 10 males with an average age of 16.9 ± 0.2 years.
- Competitive Training Group (CTG): 11 females with an average age of 16.0 ± 1.15 years and 9 males with an average age of 17.4 ± 1.5 years.

The research website “https://www.randomizer.org (accessed on 3 January 2016)” was used for research randomization.

The CG members were instructed not to practice any kind of physical exercise for 12 consecutive weeks and were evaluated only in the aspects described here.

The training groups underwent a 1-week training adaptation period, after which the protocols began; they were performed in a sports gymnasium, lasting 12 consecutive weeks, with sessions 4 times a week (Mondays, Tuesdays, Thursdays, and Fridays), supervised directly by the researchers.

The ATG performed intense aerobic training [24,25] on a treadmill. The intensity was calculated individually (75% of the reserve HR) and measured with a Polar^®^ pulse frequency meter. Each session lasted 30 min and was composed of a 5-min warm-up, 20 min of training, and a 5-min cool-down at the end.

The STG performed intense resistance training (85% 1 MR), including 4 sets of strength work, with 10 repetitions, leading to muscle fatigue. The individual intensity was determined by means of a maximum load test in each exercise, carried out before the beginning of the protocol, on two different days [26,27]. The choice of exercises aimed to achieve a comprehensive workout, covering the majority of muscle groups of the body and comprising supine bench press, back pull down, leg extension, leg curl, biceps curl, triceps extension, leg press, and upright row. As these are interval workouts with changes in equipment, the total time of each training session was 50 min, including a 5-min warm-up and a 5-min cool-down at the end.

The CTG performed 40 min of concurrent training, including the 5-min warm-up and 5-min cool-down, as follows: 20 min of aerobic training at the same intensity as the ATG and 20 min of resistance training in the same way as the STG. The order in which the forms of training (aerobic or resistance) were carried out alternated with each training session, aiming to equalize the priority of the use of the metabolic pathways and reserves for each form of training.

Researchers followed all participants in the trained groups, ensuring that the intensity of the exercises was maintained throughout the protocol period.

Every four weeks participants performed physical tests in order to adjust the training load/intensity. These assessments always took place 48 hours after the previous training session in order to prevent the acute effects of the exercise from interfering. It should be noted that blood collections were only carried out before and after the 12 weeks of intervention.

### 2.4. Statistical Analyses

The data were analyzed statistically in R Project for Statistical Computing software (version 3.5.1), and the figures were constructed using the GraphPad Prism 5.0 program. Initially, the Shapiro–Wilk normality test was performed to verify that the response variables followed a normal distribution, which is a presupposition of the Student’s paired *t*-test. For the variables that did not follow a normal distribution, the Wilcoxon non-parametric test was performed. The level of significance was set at 5%. Paired tests (Student’s *t*-test for WC, TC, HDL, TG, and BChE activity, and Wilcoxon test for SBP, DBP, LDL, and glycemia) were performed to verify any differences in each variable, before and after each intervention protocol. In cases where a difference was detected, a comparison between groups was performed to consider the aspects of each analyzed variable, using the Kruskal–Wallis test (completely randomized design [CRD]; that is, the experimental conditions were homogeneous), with *p* < 0.05.

## 3. Results

**Figure 1:**
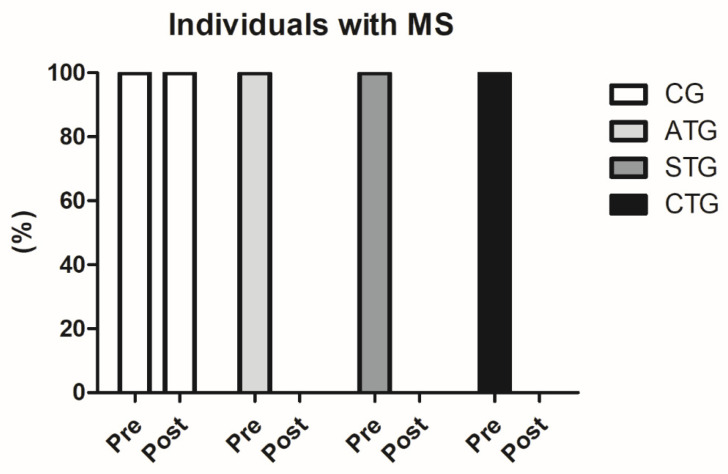
Comparisons between the number of participants classified with metabolic syndrome before and after the intervention protocol. MS: metabolic syndrome. Control Group (CG, *n* = 20), Aerobic Training Group (ATG, *n* = 20), Strength Training Group (STG, *n* = 20), and Concurrent Training Group (CTG, *n* = 20).

Prior to the intervention, 100% of the population sample (*n* = 80) were characterized with MS (WC values above 90 cm, associated with blood pressure values above 135 × 98 mmHg and HDL-c below 38 mg/dL). After 12 weeks of intervention, none of the participants who performed the training protocols were still classified as MS (*n* = 60), since blood pressure and HDL-c values were no longer within the ranges established by the IDF as standards for MS classification. This finding is extremely relevant (Figure 1).

**Figure 2:**
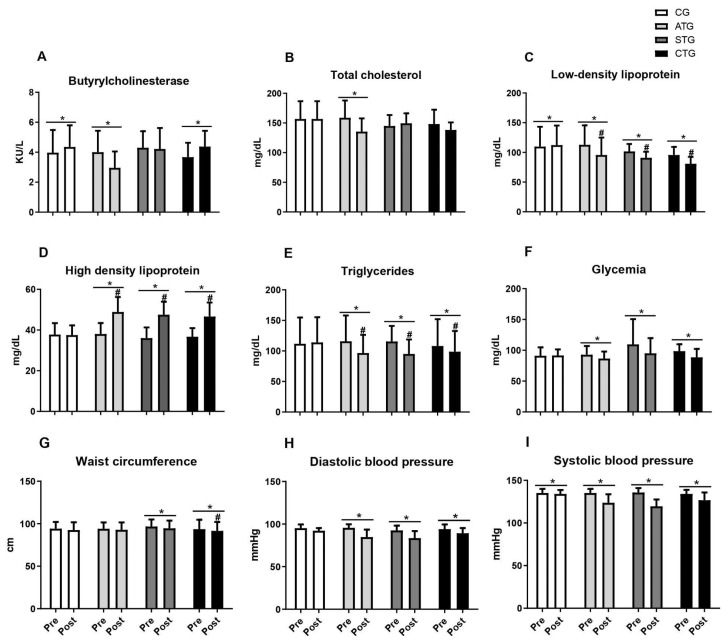
Butyrylcholinesterase values, lipid profile, blood glucose, abdominal circumference, and blood pressure of adolescents submitted to different training methods. Butyrylcholinesterase (**A**); Total cholesterol (**B**), Low-density lipoprotein (**C**), High density lipoprotein (**D**), Triglycerides (**E**); Glycemia (**F**); Waist circumference (**G**); Diastolic blood pressure (**H**); Systolic blood pressure (**I**). Pre- and post-intervention values. Control Group (CG, *n* = 20), Aerobic Training Group (ATG, *n* = 20), Strength Training Group (STG, *n* = 20), and Concurrent Training Group (CTG, *n* = 20). * different from pre-intervention in the same group. #, different from the CG at post-intervention.

Table 1 and Figure 2 present the comparisons of the parameters analyzed in relation to the pre- and post-training moments. Regarding BChE, it was observed that only aerobic training reduced serum levels after 12 weeks; concurrent training did not prevent the elevation in BChE activity after 12 weeks; and the resistance training group did not show any alteration (Table 1 and Figure 2A).

Considering total cholesterol, only aerobic training reduced serum levels (Table 1 and Figure 2B). Regarding LDL, it was observed that all the training protocols (ATG, STG, and CTG) were effective in reducing levels after 12 weeks of intervention (Table 1 and Figure 2C). Furthermore, all the training groups presented lower LDL values than the CG after 12 weeks of intervention (Figure 2C).

With respect to HDL, regardless of the training protocol, all the training groups presented increased serum levels, while no change was observed in the control group (Table 1 and Figure 2D). When comparing the aspects of HDL, the STG, ATG, and CTG were statistically equal and presented an increase in HDL in relation to the control (Figure 2D). For triglyceride levels, all the training proposals reduced the parameters, with no effect being seen in the control group (Table 1 and Figure 2E). The STG, ATG, and CTG were statistically equal and showed a reduction in TG in relation to the control (Figure 2E).

Regarding blood glucose levels, all the training proposals reduced the parameters, with no effect being seen in the control group (Table 1 and Figure 2F).

Weight training and concurrent training led to reductions in abdominal circumference measures, with no effects observed for aerobic training (Table 1 and Figure 2G). When comparing the four groups, only the STG differed from the CG (Figure 2G). Regarding blood pressure, regardless of the training protocol (ATG, STG, and CTG), both DBP (Table 1 and Figure 2H) and SBP (Table 1 and Figure 2I) were reduced after 12 weeks of intervention.

Finally, when all the variables were analyzed, segmented by group, all the training groups presented satisfactory results in relation to the majority of the analyzed items, highlighting the ATG, which presented positive results in eight of the nine variables.

## 4. Discussion

Metabolic syndrome (MS) results from an abnormal metabolism, caused by a poor diet and a lack of physical activity during childhood and adolescence [3]. Regular physical exercise is the main therapeutic strategy for preventing health problems [5]. From this perspective, the current research compared the effects of different physical exercise protocols on biomarkers in a population of adolescents. BChE can modulate lipid metabolism, affecting the concentration and nature of circulating lipids. Increased levels of BChE may be correlated with a greater release of free fatty acids into the bloodstream; thus, they affect the concentration and nature of the circulating lipids [10].

In relation to possible biomarkers, BChE has been strongly associated with MS in adolescents [28]. BChE can be considered a secondary marker for obesity-related risk factors that respond to aerobic exercise; this fact was also confirmed in this study [15].

After applying the protocols proposed by this research, it was observed that the intervention with aerobic exercises reduced BChE activity. This group also presented the most significant correlations with the other variables investigated.

Similar results were found in the study by Milano et al. [15], who evaluated the activity of the BChE enzyme in 24 overweight adolescents after 12 weeks of guided aerobic exercise, reporting a reduction in this variable, TG, and glycemia. The authors affirmed that there is a relationship between BChE activity, lipid metabolism, and metabolic stress present in obesity; this fact was corroborated in the studies by Furtado-Alle et al. [29], Boberg et al. [30], and Kálmán et al. [14]. The latter study correlated the increase in BChE activity with disorders associated with obesity, verifying an inverse relationship: when there is a reduction in BMI, TG, and LDL, there is a reduction in BChE enzymatic activity.

Some studies have verified the acute effect of aerobic exercise on BChE activity. Because BChE hydrolyses fatty acid into desacyl ghrelin and other hormones responsible for appetite, the enzyme was recently investigated as a ghrelin-suppressing factor during acute aerobic exercise and in the postprandial period. This hypothesis could contribute as a strategy to reduce nutritional intake and aid weight loss [31,32].

Dorling et al. [31] investigated individuals with a genotype associated with obesity; these individuals exhibited higher concentrations of postprandial fatty acids. An acute aerobic 1-h training protocol at an intensity of 70% VO_2_ peak reduced BChE activity and fatty acids in individuals at risk for obesity and did not increase post-exercise intake.

In another experiment, young men were tested in exercise and meal conditions. The 30-min running protocol, also at 70% of VO_2_ peak, did not change BChE activity before and after effort or meals, suggesting that BChE is not changed by acute aerobic exercises [32].

Even though a reduction in systolic and diastolic blood pressure resulting from resistance exercise has been found in a younger population (9.3 to 15.9 years), variables such as the heterogeneity of the exercise intensity, volume, frequency, or duration make it difficult to clarify the dose-response effects of this type of model in apparently healthy young people or in clinical conditions [33].

The findings of the current study show that although the experimental conditions were analogous, BChE activity demonstrated different responses.

The BChE activity response in the groups submitted to resistance and concurrent training showed no improvement, suggesting that this variable does not respond to anaerobic exercise. No studies with this analysis were found that employed this type of exercise.

In relation to the lipid variables, the current study found that ATG likewise led to better results, since it positively impacted all the variables analyzed (TC, LDL, HDL, and TG).

These results agree with those presented in the study by Javaherian et al. [34], which was based on a broad review of systematic studies and meta-analyses. The authors verified the effects of different types of exercise to manage lipid profile abnormalities in cardiac patients, showing significant improvements after aerobic and resistance exercise programs at different intensities, durations, and frequencies.

Studies corroborate the results of the present research, demonstrating that physical exercises can be used as therapeutic adjuvants in the control of lipoproteins. In a meta-analysis addressing 15 studies, the authors suggested that the intensity and the type of exercise influence the results, with high-intensity aerobic exercise possibly being the most effective [19].

Contradictions regarding the efficiency of resistance exercises in controlling the lipid profile in overweight and obese children have been reported, suggesting that this vagueness occurs due to the lack of methodological criteria and the low number of studies, making it difficult to establish a consensus about the metabolic effect of lipids [34].

This situation also occurs in relation to concurrent training, which combines aerobic with resistance exercises. The methods are quite distinct, making comparisons impossible. In 2009, for example, a study was conducted in 28 healthy young people for 16 weeks, using concurrent training and aerobic training to analyze possible impacts on the lipid profile. The results showed a reduction in LDL concentrations and an elevation in HDL that were exactly equal to the data from the group that underwent only aerobic training, indicating that the increase in resistance training in the aerobic protocol (which characterizes concurrent training) did not have a positive impact [35]. 

Moreover, a meta-analysis of 12 studies comparing aerobic exercise alone with aerobic plus resistance exercise (concurrent) observed that this model resulted in greater reductions in body mass and increases in lean mass, as well as a modulation of the metabolic profile and inflammatory status of the pediatric population, obtaining better effects in long-term programs [36]. To reach a consensus on the real effect of concurrent training, specific guidelines need to be discussed so that they can be implemented in scientific research and, subsequently, in clinical practice.

More recent studies have assessed the comparative effects of exercise programs in overweight children and adolescents, and improvements have been identified in the metabolic health parameters, regardless of the model. However, the specific exercise intensity needs to be better clarified [36,37,38].

In relation to the blood pressure values, a significant reduction was observed in all the groups who underwent the different types of training.

The hypotensive effect promoted by exercise is widely reported in the literature in normotensive and hypertensive individuals, due to its ability to facilitate autonomic, hormonal, and hemodynamic adjustments and adaptations [34], which provides evidence that the reduction in blood pressure occurs regardless of the exercise model proposed [35,39]. However, most studies identify the effectiveness of aerobic exercise in the pediatric population at metabolic risk [40,41,42].

Even though a reduction in systolic and diastolic blood pressure resulting from resistance exercise has been found in a younger population (9.3 to 15.9 years), variables such as the heterogeneity of exercise intensity, volume, frequency, or duration make it difficult to clarify the dose-response effects of this type of model in apparently healthy young people or in clinical conditions [43].

Although the hypotensive effects of resistance training are still controversial, this model, like mixed training, warrants further investigation.

Although measurements of training load/intensity adjustments were recorded every 4 weeks, blood samples were only taken before and after the 12 weeks of intervention. It should be stressed that this was because the aim of the study was to assess the effects of the 12-week physical training protocols and not shorter interventions, with load adjustments being performed every 4 weeks only for progression of the periodization originally proposed for all the protocols.

Although insulin levels were not measured and the homeostasis model assessment (HOMA) was not calculated, the authors followed the IDF criteria for MS. Furthermore, it is important to note that the study included a large sample of participants, which strengthens the reliability of the results obtained. These findings are promising and indicate the potential effectiveness of different models of physical exercise in improving indicators of MS.

## 5. Conclusions

Finally, considering the main objective of this research, it was verified that all the training groups presented improvements in most of the analyzed indicators, providing evidence of the importance of regular physical exercise as an efficient and low-cost intervention in the MS indicators. In the comparison between types of training (aerobic, resistance, or concurrent), aerobic training demonstrated efficiency in relation to the improvement in the majority of the analyzed variables. The aerobic model promoted changes in BChE activity, in contrast to all the other groups. It is suggested that further research be carried out with resistance and concomitant exercises and that the methodologies should be standardized to enable comparisons.

## Figures and Tables

**Table 1:** Mean values of BChE, TC, LDL, HDL, TG, glycemia, WC, SBP, and DBP, pre- and post-intervention.

Variables	CG			ATG			STG			CTG		
	Pre	Post	*p*	Pre	Post	*p*	Pre	Post	*p*	Pre	Post	*p*
BChE (KU/L)	3.97 ± 1.51	4.35 ± 1.45	<0.01	4.00 ± 1.44	2.96 ± 1.09	<0.01	4.30 ± 1.10	4.22 ± 1.40	0.85	3.67 ± 0.96	4.38 ± 1.05	0.03
TC (mg/dL)	156.8 ± 30.0	156.8 ± 29.9	1.00	158.8 ± 29.0	135.4 ± 22.3	<0.01	144.9 ± 18.5	149.4 ± 16.8	0.40	148.2 ± 24.2	138.2 ± 12.7	0.11
LDL (mg/dL)	109.8 ± 33.4	112.2 ± 33.0	0.02	112.7 ± 32.9	95.5 ± 29.38	<0.01	101.5 ± 12.7	90.9 ± 10.1	<0.01	95.6 ± 13.7	80.9 ± 11.7	<0.01
HDL (mg/dL)	37.8 ± 5.6	37.5 ± 4.8	0.56	38.0 ± 5.4	48.9 ± 7.3	<0.01	36.0 ± 5.2	47.5 ± 6.4	<0.01	36.7 ± 4.2	46.6 ± 6.9	<0.01
TG (mg/dL)	111.8 ± 42.9	113.9 ± 41.3	0.26	115.7 ± 42.3	96.5 ± 29.7	0.04	115.4 ± 25.5	95.0 ± 23.7	<0.01	108.1 ± 43.9	98.8 ± 33.7	0.03
Glycemia (mg/dL)	91.1 ± 13.6	91.6 ± 9.8	0.91	92.8 ± 14.0	86.5 ± 11.4	<0.01	109.6 ± 40.9	95.0 ± 24.7	<0.01	98.5 ± 11.1	88.5 ± 13.8	<0.01
WC (cm)	94.2 ± 7.9	92.5 ± 9.2	0.11	94.0 ± 7.5	92.8 ± 8.8	0.20	96.7 ± 8.2	94.5 ± 9.1	<0.01	93.5 ± 11.1	91.6 ± 10.4	<0.01
DBP (mmHg)	95.2 ± 4.3	92.2 ± 3.0	0.38	95.5 ± 4.2	84.7 ± 8.8	<0.01	92.5 ± 5.7	83.5 ± 8.2	<0.01	94.0 ± 5.5	89.2 ± 6.1	<0.01
SBP (mmHg)	135.2 ± 4.6	134.1 ± 4.6	0.02	135.2 ± 4.7	123.7 ± 9.9	<0.01	135.7 ± 5.1	119.5 ± 8.0	<0.01	134 ± 4.7	126.7 ± 9.0	<0.01

Legend: CG: Control Group; ATG: Aerobic Training Group; STG: Strength Training Group; CTG: Concurrent Training Group; BChE: butyrylcholinesterase; TC: total cholesterol; LDL: low-density lipoprotein; HDL: high-density lipoprotein; TG: triglycerides; WC: waist circumference; SBP: systolic blood pressure; DBP: diastolic blood pressure.

## Data Availability

Restrictions apply to the datasets. The datasets presented in this article are not easily available because the database is considered the intellectual property of the university proposing the study and access to the data is not available for public consultation. Requests for access to the datasets should be addressed to the corresponding author.
